# Prognostic factors for lung transplant recipients focusing on age and gender: the Japanese lung transplantation report 2022

**DOI:** 10.1007/s00595-023-02686-w

**Published:** 2023-04-19

**Authors:** Hisashi Oishi, Yoshinori Okada, Masaaki Sato, Jun Nakajima, Daisuke Nakajima, Takeshi Shiraishi, Toshihiko Sato, Takashi Kanou, Yasushi Shintani, Kentaroh Miyoshi, Shinichi Toyooka, Sumiko Maeda, Masayuki Chida, Keitaro Matsumoto, Takeshi Nagayasu, Hidemi Suzuki, Ichiro Yoshino, Yasushi Matsuda, Yasushi Hoshikawa, Hiroshi Date

**Affiliations:** 1 Department of Thoracic Surgery, Institute of Development, Aging and Cancer, Tohoku University, 4-1 Seiryomachi, Aobaku, Sendai, 980-8575 Japan; 2 Department of Thoracic Surgery, The University of Tokyo, Tokyo, Japan; 3 Department of Thoracic Surgery, Kyoto University, Kyoto, Japan; 4 Department of General Thoracic, Breast and Pediatric Surgery, Fukuoka University School of Medicine, Fukuoka, Japan; 5 Department of General Thoracic Surgery, Osaka University Graduate School of Medicine, Osaka, Japan; 6 Department of General Thoracic Surgery and Breast and Endocrinological Surgery, Okayama University Graduate School of Medicine, Dentistry and Pharmaceutical Sciences, Okayama, Japan; 7 Department of General Thoracic Surgery, Dokkyo Medical University, Mibu, Japan; 8 Department of Surgical Oncology, Nagasaki University Graduate School of Biomedical Science, Nagasaki, Japan; 9 Department of General Thoracic Surgery, Chiba University Graduate School of Medicine, Chiba, Japan; 10 Department of Thoracic Surgery, Fujita Health University School of Medicine, Toyoake, Japan

**Keywords:** Lung transplantation, Age, Gender, End-stage lung disease

## Abstract

**Purpose:**

To clarify the impact of donor and recipient characteristics on the survival of recipients before and after lung transplantation in the Japanese population.

**Methods:**

Patients’ data were collected for retrospective analysis from all authorized lung transplant centers in Japan. We included 1963 patients listed for lung transplantation by the end of December 2021, comprised of 658 deceased-donor and 270 living-donor lung transplants.

**Results:**

Primary disease had a significant impact on the mortality of patients waiting for transplantation. The indications for transplant significantly affected the post-transplant survival rate of deceased-donor lung transplant recipients. The recipient’s age also significantly affected the post-transplant survival rate of the deceased-donor and living-donor lung transplant recipients. The recipients of grafts transplanted from donors aged 61 years or older showed a worse post-transplant survival rate (≧60 years old). The survival rate for the combination of a female donor to a male recipient among the deceased-donor lung transplant recipients was the worst among the four combinations.

**Conclusion:**

The donor and recipient characteristics significantly impacted the survival of recipients after lung transplantation. The underlying mechanism of the negative impact of the gender mismatch of female donor to male recipient on post-transplant survival needs to be investigated further.

## Introduction

Lung transplantation is an established treatment for end-stage lung diseases. The latest Registry Report of the International Society for Heart and Lung Transplantation (ISHLT) included 67,493 adult lung transplant procedures from deceased donors [1]. According to this report, even as recently as January, 2010–June, 2018, most lung transplantations in the world were being performed in Europe (36.6%) and North America (54.9%), with only 8.5% being performed in other countries. In Japan, clinical lung transplantation began 15 years after the first clinical lung transplantations in North America and Europe. However, following enforcement of the Organ Transplantation Law in 1997, the first living-donor lobar lung transplantation and the first deceased-donor lung transplantations were performed in Japan in 1998 and 2000, respectively. As of December 2021, 658 deceased-donor and 270 living-donor lung transplant procedures had been performed [2] in Japan, with the number of procedures increasing since the Japanese Organ Transplantation Law was revised in 2010. The total number of deceased-donor and living-donor lung transplantations in 2021 was 93, which is a record-high according to the latest registry report on the website of The Japanese Society of Lung and Heart–Lung Transplantation [2]. At the time of introduction of lung transplantation in Japan, there were four lung transplant centers and now there are ten centers authorized to perform lung transplantation.

The Japanese Society of Lung and Heart–Lung Transplantation has been reporting the annual registry data since 2005 [3], which includes the number of lung transplant candidates registered/year, the number of lung transplants/year, indications, and survival after lung transplantations according to the procedures [3]. However, the impact of donor and recipient characteristics on the survival of recipients before and after lung transplantation in the Japanese population has not been recorded as a nationwide report. Therefore, we sought to clarify the influence of primary diseases on the survival of the patients on the waitlist. We also examined the impact of indications, transplant era, donors’ and recipients’ age, and donor-recipient gender matching on the survival of recipients after lung transplantation.

## Methods

### Ethical statement

The Ethics Committee of Tohoku University Graduate School of Medicine approved this retrospective study (No. 2021-1-061) and waived the requirement for informed consent. The IRB of each authorized lung transplant center also approved the study, which was conducted in accordance with the 1964 Declaration of Helsinki and its later amendments. The use of the donor data for this research was approved by the IRB of the Japan Organ Transplant Network (JOTN).

### Patient population and data collection

By the end of December, 2021, a total of 1963 patients had been listed for lung transplantation in Japan, with 477 candidates on the waitlist. The patients’ data were collected from all authorized lung transplant centers in Japan: Tohoku University, Dokkyo Medical University, University of Tokyo, Chiba University, Fujita Health University, Kyoto University, Osaka University, Okayama University, Fukuoka University, and Nagasaki University. By the end of December 2021, 658 deceased-donor lung transplants and 270 living-donor lobar lung transplants had been performed. The clinical data of the post-transplant recipients were also collected from all authorized lung transplant centers. The information on each deceased donor, provided by JOTN to every transplant center at the time of the transplantation, was also collected. The data on hybrid lung transplantation; namely, deceased-donor lung transplantation combined with living-donor lung transplantation, were collected as a part of the data on deceased-donor lung transplants. Because of the small number of cases, data on heart–lung transplants have not been included in this report.

### Statistical analysis

The Prism 5 (GraphPad Software Inc., La Jolla, CA) and the JMP Pro 16 statistical software (SAS Institute, Cary, NC, USA) were used to perform the statistical analyses. Patients’ overall survival was estimated using the Kaplan–Meier method, groups were compared using the log-rank test, and post hoc comparisons were done using the Holm-Sidak multiple comparison test. Linear regression was used to describe associations between donor and recipient age in deceased-donor lung transplantation. Values of *p* < 0.05 were regarded as significant.

## Results

### Candidates for lung transplantation on the waitlist as of December 2021

Among the 1963 patients listed for lung transplantation, 740 (37.7%) patients died while on the waitlist. As of December 2021, 477 candidates were waiting for lung transplantation. Table 1 shows the number of patients on the waitlist based on their disease. Both active and inactive candidates were included. The percentage of patients with interstitial pneumonia (IP) was the highest (n = 198, 41.5%), followed by pulmonary hypertension (PH, n = 107, 22.4%). Forty-eight patients (10.1%) had lymphangioleiomyomatosis (LAM), which is one of the major indications for lung transplantation in Japan. The median ages of the patients with IP and chronic obstructive pulmonary disease (COPD) were 53 and 48, respectively, with interquartile ranges (IQR) of 46–57 and 29–53, respectively. The median ages of both the IP and COPD patients were relatively higher than those of patients on the waitlist with other diseases. Conversely, the median ages of the patients with chronic lung allograft dysfunction (CLAD) and PH were lower among those on the waitlist (CLAD: median age, 28; IQR, 19–43; PH: median age, 30; IQR, 22–38). The median waiting time for all patients on the waitlist was 610 days (IQR, 304–1183 days). The median waiting time for the patients with LAM was 1679 days (IQR, 568–3054 days), which was the longest among the diseases. The second longest waiting time was 1018 days (IQR, 482–1813 days) for patients with PH. This might be because some patients with these diseases were registered on the waitlist before the introduction of specific drug therapies. For example, epoprostenol for PH and sirolimus for LAM were approved for cover by medical insurance in 1999 and 2010, respectively. All patients with LAM were female and the ratio of female patients with PH was also high (73.8%).

**Table 1:** Patients on the waitlist for lung transplantation based on their disease (as of December, 2021; n = 477)

Disease	N	%	Age, years, median (IQR^a^)		Waiting time, days, median (IQR)		Gender (male/female)			
IP^b^	198	41.5	53	(46–57)	491	(261–798)	115	–	83	(58.1%/41.9%)
LAM^c^	48	10.1	39	(34–46)	1679	(568–3054)	0	–	48	(0%/100%)
PH^d^	107	22.4	30	(22–38)	1018	(482–1813)	28	–	79	(26.2%/73.8%)
COPD^e^	24	5.0	48	(29–53)	583	(416–799)	16	–	8	(66.7%/33.3%)
BE and DPB^f^	26	5.5	43	(37–51)	540	(199–1218)	12	–	14	(46.2%/53.8%)
Lung dysfunction after HSCT^g^	30	6.3	39	(30–44)	496	(279–753)	19	–	11	(63.3%/36.7%)
CLAD^h^	9	1.9	28	(19–43)	424	(406–632)	7	–	2	(77.8%/22.2%)
Others	35	7.3	41	(30–51)	748	(320–1073)	16	–	19	(45.7%/54.3%)
Total	477		45	(33–53)	610	(304–1183)	213	–	264	(44.7%/55.3%)

*IQR* interquartile range, *IP*, interstitial pneumonia, *LAM* lymphangioleiomyomatosis, *PH* pulmonary hypertension, *COPD* chronic obstructive pulmonary disease. *BE and DPB* bronchiectasis and diffuse panbronchiolitis, *Lung dysfunction after HSCT* lung dysfunction after hematopoietic stem cell transplantation, *CLAD* chronic lung allograft dysfunction

### Survival of patients on the waitlist based on their disease

**Fig. 1:**
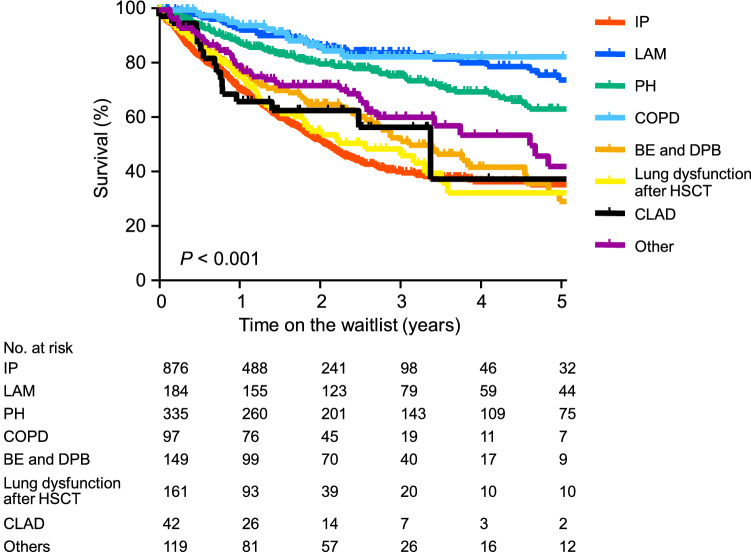
Survival of patients on the waitlist based on their disease. In total, 1963 patients were listed for lung transplantation between August, 1996 and the end of December, 2021. The 1 year, 3 year, and 5 year survival rates were 70.6%, 40.6%, and 35.9% for interstitial pneumonia (IP); 92.5%, 84.2%, and 74.3% for lymphangioleiomyomatosis (LAM); 87.7%, 75.6%, and 63.5% for pulmonary hypertension (PH); 94.2%, 82.7%, and 82.7% for chronic obstructive pulmonary disease (COPD); 77.0%, 53.1%, and 29.6% for bronchiectasis and diffuse panbronchiolitis (BE and DPB); 75.5%, 49.0%, and 32.8% for lung dysfunction after hematopoietic stem cell transplantation (lung dysfunction after HSCT); 66.4%, 56.7%, and 37.8% for chronic lung allograft dysfunction (CLAD); and 79.4%, 60.6%, and 42.4% for others, respectively. There was a significant difference (*p* < 0.001)

Figure 1 shows the survival of the 1963 patients listed for lung transplantation between August, 1996 and December, 2021. The 1 year, 3 year, and 5 year survival rates were 70.6%, 40.6%, and 35.9% for IP; 92.5%, 84.2%, and 74.3% for LAM; 87.7%, 75.6%, and 63.5% for PH; 94.2%, 82.7%, and 82.7% for COPD; 77.0%, 53.1%, and 29.6% for bronchiectasis and diffuse panbronchiolitis (BE and DPB); 75.5%, 49.0%, and 32.8% for lung dysfunction after hematopoietic stem cell transplantation (HSCT); 66.4%, 56.7%, and 37.8% for CLAD; and 79.4%, 60.6%, and 42.4% for others, respectively. The differences were significant (*p* < 0.001).

### Survival of deceased-donor lung transplant recipients based on the indication for transplant

**Fig. 2:**
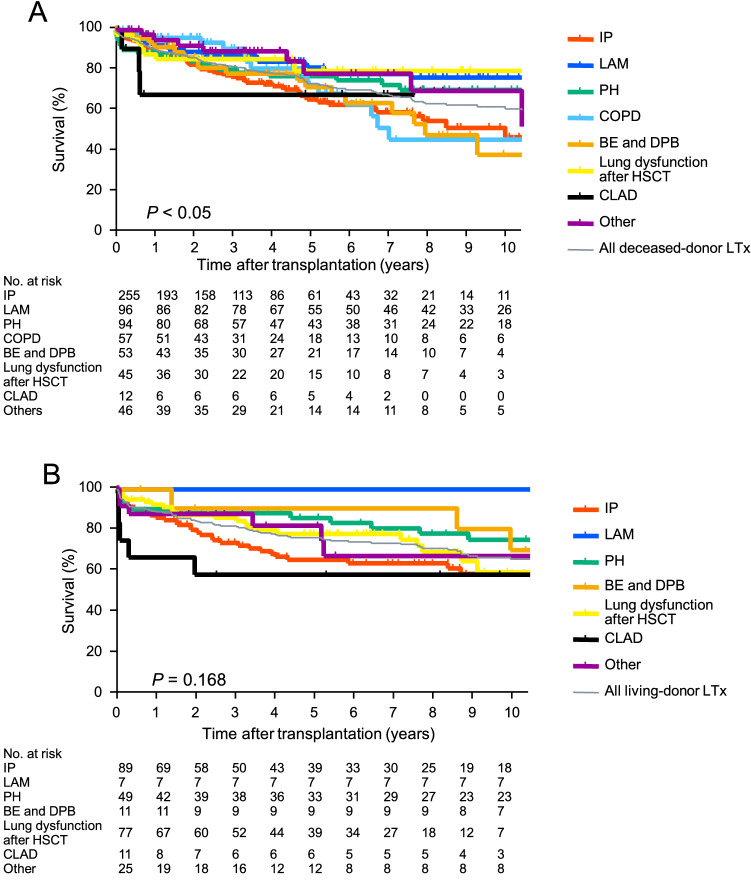
Survival of deceased- and living-donor lung transplant recipients based on the indication for transplant. **A** The survival of deceased-donor lung transplant recipients (n = 658) based on the indication for transplant is shown. The 3 year, 5 year, and 10 year survival rates were 77.1%, 65.6%, and 47.3% for interstitial pneumonia (IP); 88.2%, 81.6%, and 76.7% for lymphangioleiomyomatosis (LAM); 80.6%, 77.5%, and 70.6% for pulmonary hypertension (PH); 90.9%, 73.0%, and 46.1% for chronic obstructive pulmonary disease (COPD); 78.7%, 71.9%, and 38.7% for bronchiectasis and diffuse panbronchiolitis (BE and DPB); 85.8%, 80.1%, and 80.1% for lung dysfunction after hematopoietic stem cell transplantation (lung dysfunction after HSCT); and 89.6%, 78.6%, and 69.9% for others, respectively. The 3 year and 5 year survival rates for chronic lung allograft dysfunction (CLAD) were 68.2% and 68.2%, respectively. (The observation period of the recipients for CLAD had not yet reached 10 years at the time of analysis.) There was a significant difference (*p* < 0.05). The 3 year, 5 year, and 10 year survival rates for all deceased-donor lung transplant (LTx) recipients were 82.2%, 73.7%, and 61.4%. The survival rate of the deceased-donor lung transplant recipients for LAM was significantly better than that for recipients for IP or BE and DPB. **B** The survival of the living-donor lung transplant recipients (n = 270) based on the indication is shown. The 3 year, 5 year, and 10 year survival rates were 71.4%, 62.3%, and 55.0% for IP; 100.0%, 100.0%, and 100.0% for LAM; 87.2%, 84.7%, and 73.1% for PH; 90.0%, 90.0%, and 67.5% for BE and DPB; 84.9%, 76.2%, and 56.0% for lung dysfunction after HSCT; 54.5%, 54.5%, and 54.5% for CLAD; and 86.7%, 80.5%, and 64.4% for others, respectively. There was a significant difference (*p* = 0.168). The 3 year, 5 year, and 10 year survival rates for all living-donor LTx recipients were 80.0%, 74.1%, and 62.7%, respectively

Figure 2A shows the survival of the deceased-donor lung transplant recipients (n = 658) based on the indication for transplant. The 3 year, 5 year, and 10 year survival rates were 77.1%, 65.6%, and 47.3% for IP; 88.2%, 81.6%, and 76.7% for LAM; 80.6%, 77.5%, and 70.6% for PH; 90.9%, 73.0%, and 46.1% for COPD; 78.7%,71.9%, and 38.7% for BE and DPB; 85.8%, 80.1%, and 80.1% for lung dysfunction after HSCT; and 89.6%, 78.6%, and 69.9% for others, respectively. The 3- and 5 year survival rates for CLAD were both 68.2%. (The observation period of the recipients for CLAD has not reached 10 years). There was a significant difference (*p* < 0.05). The 3 year, 5 year, and 10 year survival rates for all deceased-donor lung transplant recipients were 82.2%, 73.7%, and 61.4%, respectively. The survival rate of the deceased-donor lung transplant recipients for LAM was significantly better than that of those for IP or BE and DPB (*p* < 0.05).

### Survival of living-donor lung transplant recipients based on the indication for transplant

Figure 2B shows the survival of the living-donor lung transplant recipients (n = 270) based on the indication. The 3 year, 5 year, and 10 year survival rates were 71.4%, 62.3%, and 55.0% for IP; 100.0%, 100.0%, and 100.0% for LAM; 87.2%, 84.7%, and 73.1% for PH; 90.0%, 90.0%, and 67.5% for BE and DPB; 84.9%, 76.2%, and 56.0% for lung dysfunction after HSCT; 54.5%, 54.5%, and 54.5% for CLAD; and 86.7%, 80.5% and 64.4% for others, respectively. There were no significant differences among these groups (*p* = 0.168). The 3 year, 5 year, and 10 year survival rates for all living-donor lung transplant recipients were 80.0%, 74.1% and 62.7%, respectively.

### Survival of deceased-donor lung transplant recipients based on the recipient age

**Fig. 3:**
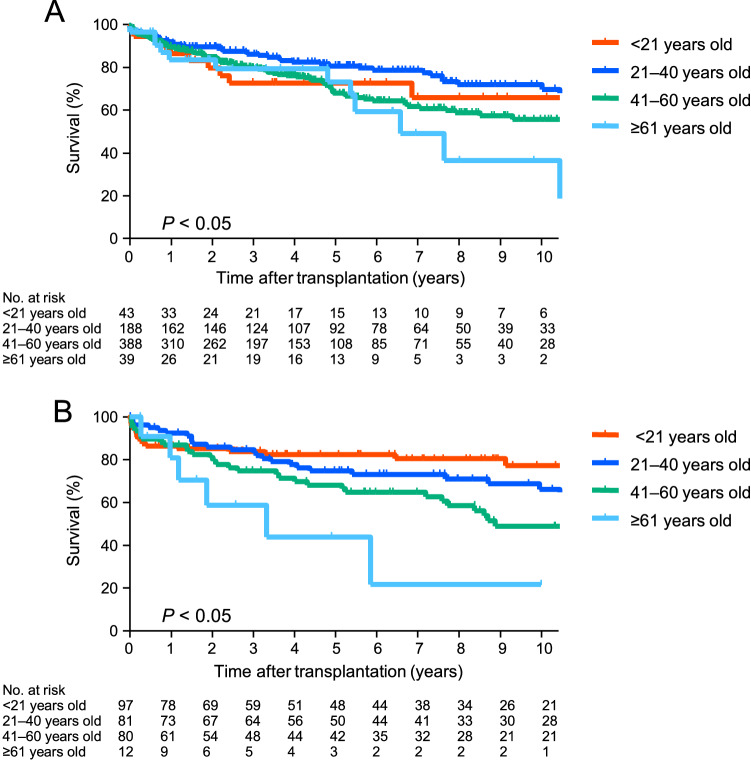
Survival of deceased- and living-donor lung transplant recipients based on the recipient age. **A** The survival of the deceased-donor lung transplant recipients (n = 658) based on the recipient’s age is shown. The 3 year, 5 year, and 10 year survival rates were 73.7%, 73.7%, and 66.9% for the recipients younger than 21 years old (< 21 years old); 87.1%, 81.6%, and 70.7% for the recipients aged 21 years or older but younger than 41 years old (21–40 years old); 80.6%, 68.9%, and 56.8% for the recipients aged 41 years or older but younger than 61 years (41–60 years old); and 80.3%, 74.1%, and 37.7% for the recipients aged 61 years or older (≧61 years old), respectively. There was a significant difference (*p* < 0.05). The survival rate of the deceased-donor lung transplant recipients aged 21–40 years old was significantly better than that of the recipients aged 41–60 years old or ≧61 years old (*p* < 0.05). **B** The survival of the living-donor lung transplant recipients (n = 270) based on the recipient age is shown. The 3 year, 5 year, and 10 year survival rates were 84.0%, 82.5%, 77.3% for the recipients aged < 21 years old; 84.6%, 74.9%, and 66.4% for the recipients aged 21–40 years old; 74.8%, 68.3%, and 49.2% for the recipients aged 41–60 years old; and 58.9%, 44.2%, and 22.1% for the recipients aged ≧ 61 years old, respectively. There was a significant difference (*p* < 0.05). The survival rate of the living-donor lung transplant recipients aged < 21 years old was significantly better than that of the recipients aged 41–60 years old or those aged ≧61 years old (*p* < 0.05). The survival rate of the living-donor lung transplant recipients aged 21–40 years old was significantly better than that of the recipients aged ≧ 61 years old (*p* < 0.05)

Figure 3A shows the survival of the deceased-donor lung transplant recipients (n = 658) based on the recipient age. The 3 year, 5 year, and 10 year survival rates were 73.7%, 73.7%, and 66.9% for the recipients younger than 21 years old (< 21 years old); 87.1%, 81.6% and 70.7% for the recipients aged 21 years or older but younger than 41 years (21–40 years old); 80.6%, 68.9%, and 56.8% for the recipients aged 41 years or older but younger than 61 years (41–60 years old); and 80.3%, 74.1%, and 37.7% for the recipients aged 61 years or older (≧61 years old), respectively. There were significant differences (*p* < 0.05). The survival rate of the deceased-donor lung transplant recipients at 21–40 years old was significantly better than those of recipients at 41–60 or ≧61 years old (*p* < 0.05).

### Survival of living-donor lung transplant recipients based on the recipient age

Figure 3B shows the survival of the living-donor lung transplant recipients (n = 270) based on their age. The 3 year, 5 year, and 10 year survival rates were 84.0%, 82.5%, and 77.3% for the recipients aged < 21 years old; 84.6%, 74.9%, and 66.4% for the recipients aged 21–40 years old; 74.8%, 68.3%, and 49.2% for the recipients aged 41–60 years old; and 58.9%, 44.2% and 22.1% for the recipients aged ≧61 years old, respectively. There were significant differences (*p* < 0.05). The survival rate of the living-donor lung transplant recipients aged < 21 years old was significantly better than that of recipients aged 41–60 years old or ≧61 years old (*p* < 0.05). The survival rate of the living-donor lung transplant recipients aged 21–40 years old was significantly better than that of recipients aged ≧61 years old (*p* < 0.05).

### Survival of deceased-donor lung transplant recipients based on the year of transplant

**Fig. 4:**
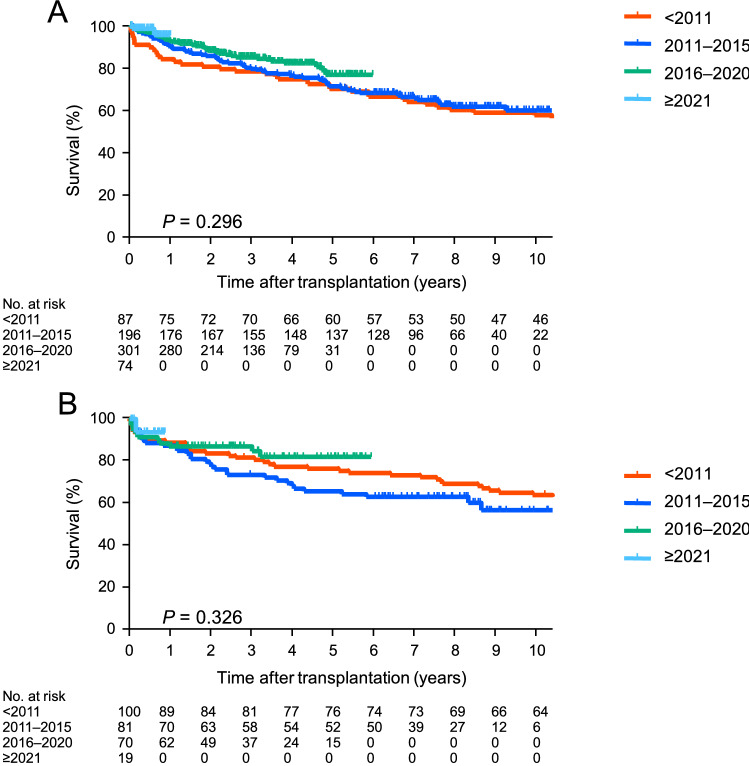
Survival of deceased- and living-donor lung transplant recipients based on transplant year. **A** The survival of the deceased-donor lung transplant recipients (n = 658) based on the transplant period is shown. The 3 year, 5 year, and 10 year survival rates were 78.2%, 69.9%, and 57.4% for the recipients transplanted before 2011 (< 2011) and 79.4%, 71.1%, and 59.7% for the recipients transplanted in 2011 or later but before 2016 (2011–2015), respectively. The 3 year and 5 year survival rates were 85.0% and 76.6% for the recipients transplanted in 2016 or later but before 2021 (2016–2020), respectively. There was no significant difference (*p* = 0.296). **B** The survival of the living-donor lung transplant recipients (n = 270) based on the transplant year is shown. The 3 year, 5 year, and 10 year survival rates were 81.6%, 76.5%, and 64.1% for the recipients transplanted before 2011 and 73.5%, 65.7%, and 56.7% for the recipients transplanted in 2011–2015, respectively. The 3 year and 5 year survival rates were 87.1% and 82.1% for the recipients transplanted in 2016–2020, respectively. There was no significant difference (*p* = 0.326)

Figure 4A shows the survival of the deceased-donor lung transplant recipients (n = 658) based on the year of transplant. The 3 year, 5 year, and 10 year survival rates were 78.2%, 69.9%, and 57.4% for the recipients transplanted before 2011 (< 2011) and 79.4%, 71.1% and 59.7% for the recipients transplanted in 2011 or later but before 2016 (2011–2015), respectively. The 3 year and 5 year survival rates were 85.0% and 76.6% for the recipients transplanted in 2016 or later but before 2021 (2016–2020), respectively. There were no significant differences among these groups (*p* = 0.296).

### Survival of living-donor lung transplant recipients based on the year of transplant

Figure 4B shows the survival of the deceased-donor lung transplant recipients (n = 270) based on the year of transplant. The 3 year, 5 year, and 10 year survival rates were 81.6%, 76.5%, and 64.1% for the recipients transplanted before 2011 and 73.5%, 65.7%, and 56.7% for the recipients transplanted in 2011–2015, respectively. The 3 year and 5 year survival rates were 87.1% and 82.1% for the recipients transplanted in 2016–2020, respectively. There were no significant differences among these groups (*p* = 0.326).

### Survival of deceased-donor lung transplant recipients based on the donor age

**Fig. 5:**
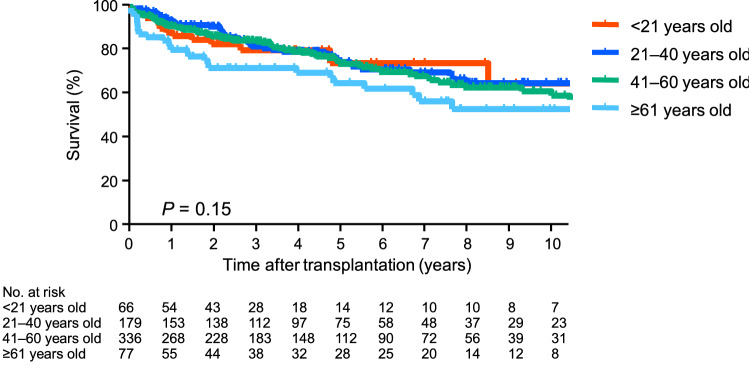
Survival of deceased-donor lung transplant recipients based on the donor age. The survival of the deceased-donor lung transplant recipients (n = 658) based on the donor age is shown. The 3 year, 5 year, and 10 year survival rates were 80.7%, 75.0%, and 65.6% for recipients transplanted from donors younger than 21 years old (< 21 years old); 82.4%, 75.3%, and 65.9% for recipients transplanted from donors aged 21 years or older but younger than 41 years (21–40 years old); 84.7%, 74.6%, and 60.1% for recipients transplanted from donors aged 41 years or older but younger than 61 years (41–60 years old): and 72.8%, 65.9%, and 53.9% for recipients transplanted from donors aged 61 years or older (≧61 years old), respectively. There was no significant difference (*p* = 0.15). When we compared the survival rates of recipients transplanted from donors younger than 61 years old with those from donors ≧ 61 years old, there was a significant difference between the age groups (*p* < 0.05). The 3 year, 5 year, and 10 year survival rates were 83.4%, 74.8%, and 62.5% for the recipients transplanted from donors ≧60 years old, respectively

Figure 5 shows the survival of the deceased-donor lung transplant recipients (n = 658) based on the donor age. The 3 year, 5 year, and 10 year survival rates were 80.7%, 75.0%, and 65.6% for the recipients transplanted from donors aged < 21 years old; 82.4%, 75.3%, and 65.9% for the recipients transplanted from donors aged 21–40 years old; 84.7%, 74.6%, and 60.1% for the recipients transplanted from donors aged 41–60 years old; and 72.8%, 65.9%, and 53.9% for the recipients transplanted from donors aged ≧61 years old, respectively. There were no significant differences (*p* = 0.15). In fact, there were no significant differences in survival in all comparisons among the age groups. On the other hand, when we compared the survival rates for the recipients transplanted from donors younger than 61 (< 61 years old) and for those from donors aged ≧61 years old, there was a significant difference between these age groups (*p* < 0.05). The 3 year, 5 year, and 10 year survival rates were 83.4%, 74.8, and 62.5% for the recipients transplanted from donors aged ≧61 years old.

### Correlation between donor and recipient age in deceased-donor lung transplantation

**Fig. 6:**
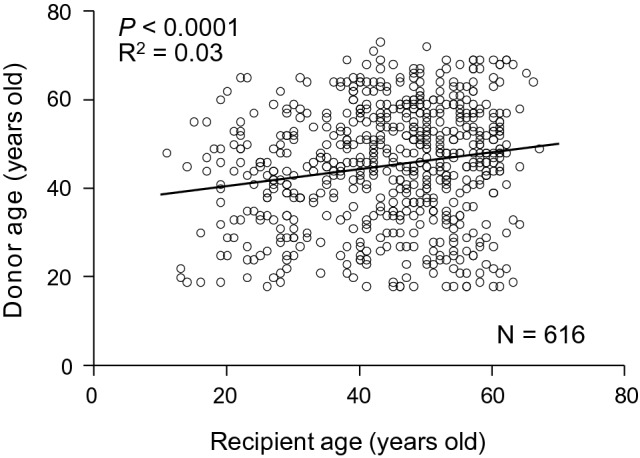
Correlation between donor and recipient age in deceased-donor lung transplantation. The correlation between donor and recipient age in deceased-donor lung transplantation is shown. We analyzed 616 deceased-donor lung transplants, the donors of which were 18 years old or older. Donor age was found to have a significant positive correlation with recipient age (*p* < 0.0001), although it was weak (R^2^ = 0.03)

Figure 6 shows the correlation between donor and recipient age in deceased-donor lung transplantation. In Japan, if the donor is under the age of 18, the lung graft is preferentially allocated to a recipient under the age of 18. Accordingly, we analyzed 616 deceased-donor lung transplants, the donors of which were 18 years or older. Donor age was found to have a significant positive correlation with recipient age (*p* < 0.0001), although it was very weak (R^2^ = 0.03).

### Survival of deceased-donor lung transplant recipients based on the donor–recipient gender matching

**Fig. 7:**
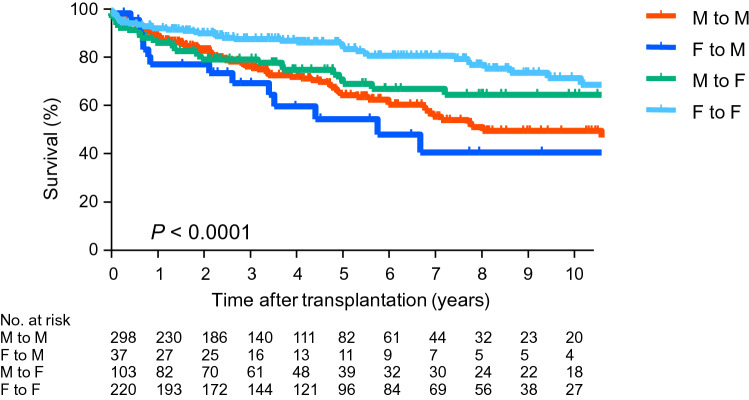
Survival of deceased-donor lung transplant recipients based on donor–recipient gender matching. Survival of the deceased-donor lung transplant recipients (n = 658) based on the donor–recipient gender matching is shown. The 3 year, 5 year, and 10 year survival rates were 77.7%, 66.6%, and 52.0% for the male donor to male recipient (M to M) combination; 71.5%, 56.8%, and 43.3% for the female donor to male recipient (F to M) combination; 81.2%, 71.2%, and 66.8% for the male donor to female recipient (M to F) combination; and 89.5%, 85.5%, and 70.9% for the female donor to female recipient (F to F) combination, respectively. There was a significant difference (*p* < 0.0001). The survival rate of the F to F combination was significantly better than that of M to M or F to M combination (*p* < 0.05)

Figure 7 shows the survival of the deceased-donor lung transplant recipients (n = 658) based on the donor–recipient gender matching. The 3 year, 5 year, and 10 year survival rates were 77.7%, 66.6%, and 52.0% for the male donor to male recipient (M to M) combination; 71.5%, 56.8%, and 43.3% for the female donor to male recipient (F to M) combination; 81.2%, 71.2%, and 66.8% for the male donor to female recipient (M to F) combination; and 89.5%, 85.5%, and 70.9% for the female donor to female recipient (F to F) combination, respectively. There was a significant difference (*p* < 0.05). The survival rate for the F to F combination was significantly better than that for the M to M and that for the F to M combinations (*p* < 0.05). The 3 year, 5 year, and 10 year survival rates were 77.0%, 65.5%, and 51.1% for the male recipients (of the M to M and the F to M combinations), and 86.9%, 81.0%, and 70.1% for the female recipients (of the M to F and F to F combinations). There was a significant difference (*p* < 0.05).

## Discussion

Long waiting times and a high mortality rate of patients on the waitlist are serious problems for Japanese lung transplantation. Primarily, the cause of this is the shortage of organ donations after brain death, with only 0.61 organ donations/million population (pmp) in Japan [4], whereas there were 41.9 pmp in the United States [5] and 9.22 pmp in Korea [4]. Significant differences in the survival of the patients on the waitlist according to disease were also apparent in this nationwide study, as identified in previous single center studies [6]. Patients with IP on the waitlist had the worst 3-year survival rate of 40.6%. The establishment of lung allocation scores, which is working effectively in the United States, is one solution, but increasing the absolute number of organ donations should be the priority in Japan.

Our analysis of the survival of deceased-donor lung transplant recipients based on the indication for transplant demonstrated that the recipients transplanted for LAM had a better survival rate than those transplanted for IP or BE and DPB. As shown in Table 1, the median age of the candidates with LAM was relatively young, which may account for their better survival rate. Indeed, the younger recipients of deceased-donor lung transplantation had a better survival rate than the older recipients (Fig. 3A). Because of the limited number of recipients aged < 21 years old and ≧61 years old, comparison of the recipients aged 21–40 years old and 41–60 years old was a useful reference, revealing a significant difference. The post-transplant survival rates for some indications were significantly improved by lung transplantation, while those of other indications were not. Some patients were registered on the waitlist before specific drug therapies were introduced, such as epoprostenol for PH and sirolimus for LAM. The systemic condition of these patients has been greatly improved by these drug therapies, and the survival rates on the waitlist are now better than for those with other indications. This may explain the slight difference between the waitlist and post-transplant survival rates.

There was no significant difference in the analysis of the survival of the living-donor lung transplant recipients based on the indication. The younger recipients of living-donor lung transplantation showed better survival rates than the older recipients of living-donor lung transplantation (Fig. 3B). Unlike the recipients of deceased-donor lung transplantation, the most common age group for living-donor lung transplant recipients was < 21 years old and this group showed a better survival rate than the 41–60 or ≧61 groups; however, there was no significant difference between the recipients aged < 21 years old and those aged 21–40 years old.

We investigated the survival rates of lung transplant recipients according to the year of the transplant. Whereas the survival rates of both deceased- and living-donor lung transplant recipients in 2016–2020 seemed better than those of other transplant periods, there was no significant difference. As described in previous reports [3, 7–9], regardless of the transplant year, the survival rates in Japan are generally better than those recorded in the ISHLT TTX Registry [10]. For example, the 5 year survival rate for adult lung transplant recipients in 2009–2016 according to the ISHLT TTX Registry was 58.1% [10], whereas the 5-year survival rate of deceased-donor lung transplant recipients in 2011–2015 was 71.1% in the present study. According to the ISHLT TTX Registry in 2020, the survival rate was lower 12 months after transplantation for recipients of organs from donors aged ≥ 50 years old [11]. In the present study, the donor age did not significantly affect the post-transplant survival rate of the deceased-donor lung transplant recipients according to stratification of the donor age: < 21, 21–40, 41–60, and ≧61 years old. However, when we compared the survival rates of recipients transplanted from donors aged ≧61 years old with those of recipients transplanted from donors < 60 years old, there was a significant difference. As the number of elderly donors was limited in this cohort, further analysis will be required.

In our analysis of the survival of deceased-donor lung transplant recipients, the post-transplant survival rate for the combination of F to M was the worst among the four gender combinations. Some previous reports of a large cohort demonstrated a similar negative impact of the F to M combination on post-transplant survival. Thabut et al. reported that the F to M combination was associated with a hazard ratio of 1.45 to the reference combination of M to M in a cohort of 785 adult lung transplant recipients [12]. In another study by Sato et al., the effects of gender combinations on recipient survival were analyzed by using the data of 9651 lung transplantation procedures in the ISHLT Registry. They demonstrated by logistic regression analysis that the combination of F to M was associated with higher early mortality, whereas the combination of F to F was associated with a lower early mortality [13]. They also found a significantly higher hazard of the F to M combination and a lower hazard of F to F by Cox proportional hazard analysis [13]. In the present study, the survival rate for female recipients (M to F and F to F combinations) was significantly better than that for male recipients (M to M and F to M combinations). This significant difference is attributed to the power of the survival of the combination of F to F (n = 220). Indeed, the combination of M to F (n = 103) was not significantly different from the other combinations. Another reason for the difference may be that LAM patients with favorable survival were included among the female recipients.

The underlying mechanism of the negative impact of the F to M combination on post-transplant survival could be related to the lung size imbalance between a female donor and male recipient. On allocating lung allografts to candidates on the waitlist in Japan, JOTN matches the predicted forced vital capacity (FVC) between the donor and the recipient. Thus, a donor with the candidate’s predicted FVC of a range from 70 to 130% is always allocated as a candidate. Although there does not seem to be a significant size mismatch between the donor and recipient in all four gender combinations, further research on the details of lung size imbalance is required. There are some reports of a negative impact of the F to M combination on the prognosis after liver and heart transplantations [14, 15]. The underlying reasons for this are still under debate. The mechanism may be biological rather than physiologic, such as graft size mismatch.

This study had several limitations. First, we did not perform multivariable analyses adjusted for potential confounders. We demonstrated important findings concerning the impact of recipient age on the survival rate in both deceased- and living-donor lung transplants, but need to clarify whether this factor persists in a multivariable analysis. For instance, since lung transplant surgeons tend to choose a donor of a similar age to the recipient, we should consider the donor age as a cofounder for a worse survival in older recipients. As far as we examined the association between donor and recipient age of deceased-donor lung transplant in the present study, the positive correlation was weak. Although the impact of donor age on recipient survival may be weak, future studies are needed. Second, the gender mismatch had an impact on post-transplant survival, and we speculate that there are biological mechanisms not yet identified.

In conclusion, the survival rate after lung transplantation in Japan is better than that recorded in the international data; however, a long waiting time and high mortality rate of patients on the waitlist remain serious problems. The indication for transplant significantly affected the post-transplant survival rate of deceased-donor lung transplant recipients in Japan; however, recipients with certain diseases are listed and transplanted at a younger age, which may have a greater effect on survival. Not surprisingly, the younger recipients showed better survival rates than the older recipients after deceased-donor and living-donor lung transplantation in this study. The recipients transplanted from donors ≧61 years old tended to have worse survival rates, although the number of elderly donors was small in this cohort. A future study is necessary to clarify whether these factors persist in a multivariable analysis. The survival rate for the combination of F to M in the deceased-donor lung transplant recipients in this study was the worst among all four gender combinations. The underlying mechanism of the negative impact of the gender mismatch of F to M on post-transplant survival also needs further study.

## Data Availability

The data produced and analyzed for this study are available from the corresponding author on reasonable request.
